# Leveraging Endogenous Dendritic Cells to Enhance the Therapeutic Efficacy of Adoptive T-Cell Therapy and Checkpoint Blockade

**DOI:** 10.3389/fimmu.2020.578349

**Published:** 2020-09-25

**Authors:** Mie Linder Hübbe, Ditte Elisabeth Jæhger, Thomas Lars Andresen, Mads Hald Andersen

**Affiliations:** ^1^National Center for Cancer Immune Therapy (CCIT-DK), Department of Oncology, Copenhagen University Hospital Herlev, Copenhagen, Denmark; ^2^Department of Health Technology, Technical University of Denmark, Kongens Lyngby, Denmark

**Keywords:** cancer immunotherapy, immune checkpoint blockade, combination therapies, T-cell therapy, dendritic cells

## Abstract

Adoptive cell therapy (ACT), based on treatment with autologous tumor infiltrating lymphocyte (TIL)-derived or genetically modified chimeric antigen receptor (CAR) T cells, has become a potentially curative therapy for subgroups of patients with melanoma and hematological malignancies. To further improve response rates, and to broaden the applicability of ACT to more types of solid malignancies, it is necessary to explore and define strategies that can be used as adjuvant treatments to ACT. Stimulation of endogenous dendritic cells (DCs) alongside ACT can be used to promote epitope spreading and thereby decrease the risk of tumor escape due to target antigen downregulation, which is a common cause of disease relapse in initially responsive ACT treated patients. Addition of checkpoint blockade to ACT and DC stimulation might further enhance response rates by counteracting an eventual inactivation of infused and endogenously primed tumor-reactive T cells. This review will outline and discuss therapeutic strategies that can be utilized to engage endogenous DCs alongside ACT and checkpoint blockade, to strengthen the anti-tumor immune response.

## Introduction

The ability of the immune system to recognize and eliminate cancer cells has paved the way for the development of cancer immunotherapies that target components of the immune system to mobilize a tumor-reactive immune response (1). Adoptive cell therapy (ACT) using T cells is an example of a cancer immunotherapy that has become a potentially curative option for subgroups of patients with melanoma and hematological malignancies (2). ACT is based on a systemic treatment with tumor-reactive autologous T cells that are obtained from tumor biopsies or blood samples, expanded *in vitro* and infused back to the patient (3, 4). This process can involve selection of tumor-reactive clones or genetic modification to generate chimeric antigen receptor (CAR) T cells or T cell receptor (TCR) modified T cells that recognize cancer-specific antigens (5). ACT using tumor infiltrating lymphocytes (TILs) is being used to treat patients with advanced stage melanoma and have mounted durable complete responses in up to 20% of treated patients (6, 7). CAR-T cells targeting the shared tumor antigen CD19 have been used to treat adult and pediatric patients suffering from B-cell acute lymphocytic leukemia (8), reaching up to 90% response rate in some clinical trials (9).

**Figure 1:**
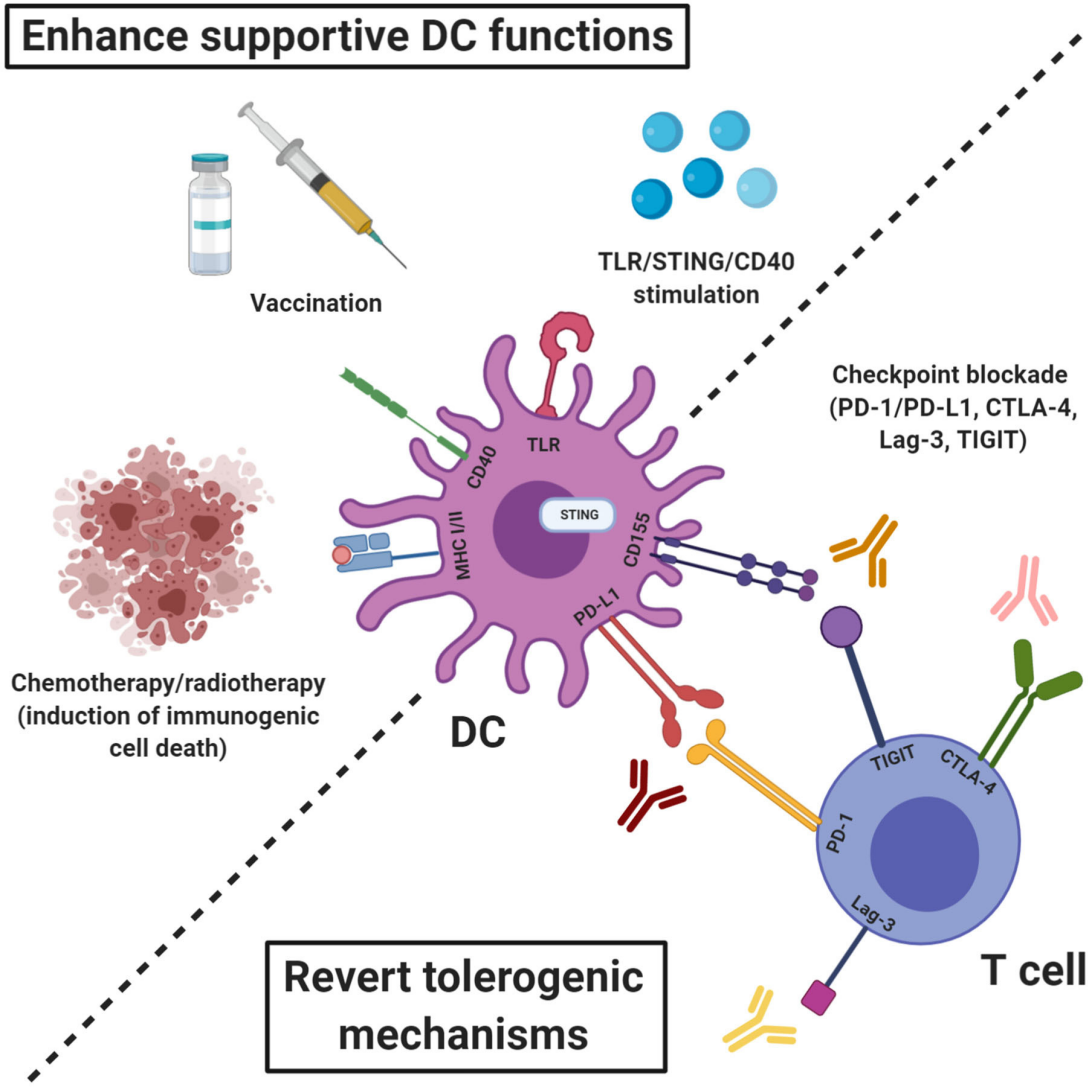
Therapeutic strategies to engage endogenous DCs alongside ACT to promote T cell priming and enhance effector functions. The therapeutic efficacy of ACT can be enhanced by induction of epitope spreading which requires tumor antigen presentation by activated DCs. The T cell priming abilities of endogenous DCs can be enhanced by promoting activation and antigen presentation e.g., through stimulation of TLRs, STING, or CD40, induction of immunogenic cell death or vaccination with tumor- or viral antigens. Eventual inactivation of infused or endogenously primed T cells by engagement of checkpoint expressed by cells of the tumor stroma can be inhibited by checkpoint blockade using antibodies targeting e.g., PD-1/PD-L1, CTLA-4, Lag-3, and TIGIT.

Clinical success of ACT has been correlated with the ability of the transferred T cells to undergo post-infusion priming and expansion, which is dependent on the phenotype of infused T cells (10–12) as well as antigen presentation and activation of dendritic cells (DCs) in the tumor-draining lymph node (tdLN) (13–15). Following priming and expansion, the therapeutic efficacy of the transferred T cells is dependent on their ability to engraft the tumor and maintain their effector functions. Thus, even sufficiently primed T cells can lose their tumor-reactivity due to escape mechanisms adapted by the tumor (16, 17), such as downregulation of the cognate antigen (18). Accordingly, it has been found that many patients treated with CAR-T cells targeting CD19 eventually suffer from relapse with CD19-negative leukemias (19, 20). Tumor escape has also been described in melanoma patients treated with TILs, where ACT was found to alter the antigenic landscape by causing target antigen downregulation (21). Relapse caused by loss of antigen can be ameliorated by the engagement of endogenous T cells to facilitate recognition of a broader tumor antigen repertoire (22–24). This phenomenon, denoted epitope spreading, is facilitated by peripheral, migratory DCs that transport antigen from the tumor to the tdLN, where naïve, endogenous tumor-reactive T cells can be primed (25) (Figure 1). Thereby the engagement of DCs alongside ACT can help to facilitate a broader and durable therapeutic response.

Another major obstacle to clinically efficient ACT is an eventual inactivation of infused and endogenously primed T cells via engagement of immune checkpoints, such as programmed cell death protein 1 (PD-1) and cytotoxic T-lymphocyte-associated protein 4 (CTLA-4), expressed by activated T cells (26). Checkpoint blockade has been a major milestone in the field of cancer immunotherapy and has shown remarkable clinical success (27). Accordingly, in 2018, the discovery that inhibition of negative immune regulation through checkpoint inhibition could be utilized for cancer therapy was awarded with the Nobel Prize jointly to James P. Allison and Tasuku Honjo (28). Immune checkpoint engagement results in an inactivation of T cells through binding of PD-1, expressed by activated T cells, to programmed death-ligand 1 (PD-L1), expressed by cells of the tumor stroma, e.g., cancer cells and other immune cells. Priming and activation of T cells can also be inhibited by interaction between CD28 on T cells and CTLA-4 expressed by regulatory T cells (Tregs) (26). In order to become activated, T cells must receive co-stimulatory signals from antigen presenting cells, such as DCs, through interaction between CD28 and B7 (CD80 or CD86) and Tregs can prevent this interaction by hijacking B7 through binding to CTLA-4, thereby blocking the binding between CD28 and B7. Antibody-based blocking of PD-1 or PD-L1 can therefore prevent inhibition of activated T cells whereas CTLA-4 blockade can enhance the priming of T cells.

Besides PD-1 and CTLA-4, other co-inhibitory receptors that influence anti-tumor immune responses have been discovered. In particular Lag-3 and T cell immunoreceptor with Ig and ITIM domains (TIGIT) are of interest in this respect and blockade of these receptors are being explored in clinical trials.

Lag-3 is upregulated by activated CD4+ and CD8+ T cells, it structurally resembles the CD4 co-receptor and binds MHC class II molecules with high affinity (29). Lag-3 is also expressed by Tregs and Lag-3 blockade has been shown to abrogate the suppressive functions of Tregs. Lag-3+ Tregs produces high amounts of IL-10 and transforming growth factor beta (TGF-β) and expand in tumor tissue of patients with melanoma and colorectal cancer (30). In pre-clinical cancer models, Lag-3 expression has been found to be co-expressed with PD-1 on tumor-infiltrating CD4+ and CD8+ T cells and co-blockade of Lag-3 and PD-1 can improve the proliferation and cytokine production of tumor-antigen specific CD8+ T cells (31). Also, Lag-3 blockade has been shown to have a synergistic therapeutic effect in combination with tumor antigen vaccination (32).

TIGIT is expressed by activated T cells, NK cells memory T cells and a subset of Tregs. TIGIT binds to CD155 and CD112 that are expressed by antigen-presenting cells (APCs), T cells and other non-hematopoietic cells, including tumor cells. Engagement of TIGIT to CD155 on DCs has been shown to inhibit IL-12p40 production and instead induce IL-10 production, thereby rendering DCs tolerogenic rather than inflammatory (33). In this way, TIGIT can indirectly inhibit the priming of tumor-reactive T cells but TIGIT engagement can also directly induce T cell inhibition by blocking T cell activation, proliferation, and acquisition of effector functions (34). In Tregs, TIGIT expression marks a phenotype that suppresses pro-inflammatory type I and type 17 responses and TIGIT engagement on Tregs has been shown to induce IL-10 secretion (35). TIGIT has been shown to be highly expressed by TILs in melanoma patients (36) and in murine tumors, the most dysfunctional TIL phenotype is CD8+ T cells that co-express TIGIT, PD-1, Tim-3, and Lag-3 (37). Consequently, in melanoma patients, co-blockade of PD-1 and TIGIT has been shown to improve proliferation, cytokine production and degranulation of CD8+ TILs (36).

The use of PD-1 and CTLA-4 blockers has been shown to increase the durable response rates and overall survival of responding patients when administered as monotherapies. However, the clinical benefits of the checkpoint blockers as monotherapies are limited by relatively low objective response rates, e.g., 10–16% for ipilimimab and 30–40% for nivolumab and pembrolizumab in metastatic melanoma patients (38–40). The combined treatment of ACT and checkpoint blockade has however been explored with encouraging results. ACT in combination with blockade of both PD-1 and CTLA-4 has shown particularly good effects (41), most likely because the anti-PD-1 treatment counteracts PD-L1-mediated inactivation of T cells whereas anti-CTLA-4 enhances priming of endogenous tumor-reactive T cells (42, 43). The combination of ACT and anti-PDL1/PD-1 or CTLA-4 might be of particular relevance as release of IFN-γ by T cells in the TME has been shown to induce checkpoint expression in both stromal cells and myeloid cell subsets, thereby constituting a negative feedback loop which hinders the continuous function of infused T cells (44, 45).

Clinical studies evaluating the efficacy of Lag-3 and TIGIT inhibition are still in early phases and despite encouraging results, the majority of patients still fail to respond to PD-1 or CTLA-4 blockade as mono- or combination therapy. This lack of sustained therapeutic responses can partly be attributed to the actions of other immune suppressive mechanisms in the tumor, e.g., secretion of amino acid depleting enzymes [such as Arginase-1 and Indolamin-2,3-dioxygenase (IDO)], TGF-β or IL-10 (46). For this reason, it can be necessary to include additional treatments that counteract these immunosuppressive mechanisms. There are several therapeutic strategies that can be utilized for this purpose, but particularly stimulation of endogenous DCs represents an interesting approach.

In this review, we will discuss strategies, that have been described pre-clinically and clinically, to improve the efficacy of ACT and immune checkpoint blockade by engaging endogenous DCs to support the effector functions of infused and endogenous tumor-reactive T cells. Our primary focus will be on CD8+ T cells and the subsets of DCs that that are essential for stimulating a tumor-reactive T cell response, i.e. cross-presenting conventional type 1 dendritic cells (cDC1s) and type I interferon (IFN) producing plasmacytoid DCs (pDCs).

## Dendritic CELL Subtypes and Their Role in T Cell Priming

In order to become activated and gain an effector phenotype, naïve T cells must to be introduced to their cognate antigen presented by activated APCs in the context of MHC molecules. DCs are the most effective type of APCs and they are indispensable for initiating an anti-tumor response (47). The outcome of an interaction between a DC and a T cell is however critically dependent on the activation status of the DC. Consequently, antigen presentation by DCs in the absence of co-stimulatory molecules or in the presence of co-inhibitory signals can result in induction of antigen-specific CD8+ T cell tolerance (48) or expansion of antigen-specific regulatory T cells (Tregs) (49, 50). The tolerogenic DCs can be induced by IL-10 that causes downregulation of co-stimulatory molecules and decreases the secretion of inflammatory chemokines (51). Priming of tumor-reactive T cells can therefore only be achieved if there is availability of tumor antigens in an inflammatory environment that also facilitates DC activation. DCs can become activated by pathogen-associated molecular patterns (PAMPs), inflammatory cytokines, and damage-associated molecular patterns (DAMPs) (52). Activation of DCs stimulates intrinsic processes necessary for T cell priming, including accumulation of MHC class I molecules in MHC loading intracellular compartments for enhanced cross-presentation (53), upregulation of co-stimulatory molecules (54) and secretion of T cell promoting cytokines (55).

Due to the pivotal role of the DC activation status on T cell priming, tumor infiltration of immature or otherwise functionally deficient DCs presents an obstacle for efficient ACT. Non-activated tumor-associated DCs can contribute to the induction of a tolerable environment that both limits the effector functions of infused T cells and affects priming of endogenous T cells (56). It is therefore relevant to discuss therapeutic strategies that can increase the availability of tumor antigens and enhance DC activation as adjuvant therapies to ACT. Enhancing the tumor antigen presentation and DC activation will not only remove a potential barrier to T cell functions, but also enhance post-infusion priming of T cells and support the mobilization of an endogenous T cell response.

### Conventional Dendritic Cells

cDCs are the most effective types of APCs and they are dedicated to the continuous sampling of antigen. Precursors to cDCs are released from the bone marrow where after they enter lymphoid organs or other peripheral sites and develop into migratory or resident DC subsets (57). The migratory DCs travel to the local lymph nodes (LNs) via the afferent lymph, where they are able to mature and function (58, 59). Both human and murine cDCs can be divided into cDC1s and cDC2s and these subtypes can be further discriminated on the basis of surface marker and transcription factor expression (Table 1). An important difference between cDC1s and cDC2s is their ability to perform cross-presentation (60), which is a process where exogenous derived antigens are internalized, processed and presented on MHC I molecules. Cross-presentation is a pre-requisite for induction of a tumor reactive cytotoxic T cell response because it enables the presentation of exogenous tumor antigens on MHC class I molecules, which are normally presented on MHC class II molecules. cDC1s are known to excel at cross-presentation of antigen to CD8+ T cells and they are the main producers of IL-12 (61, 62). In contrast, cDC2s orchestrate CD4+ T helper responses (60).

**Table 1:** Main markers of dendritic cell subtypes.

	**Human**	**Murine**
cDC1	**Surface markers** MHC II, CD11c, XCR1, CD141, CLEC9A **Transcription factors** BATF3, IRF8	**Surface markers** MHC II, CD11c, XCR1, CD8a/CD103 **Transcription factors** BATF3, IRF8
cDC2	**Surface markers** MHC II, CD11c, CD1c, CD172a **Transcription factors** IRF4	**Surface markers** MHC II, CD11c, CD11b, CD172a **Transcription factors** IRF4
pDC	**Surface markers** MHC II, CD11c, CD123, CD303, CD304 **Transcription factors** TCF4 (E2-2), BCL11A	**Surface markers** MHC II, CD11c, Siglec-H, Ly-6C **Transcription factors** TCF4 (E2-2)

In a cancer immunotherapy context, the important function of intra-tumoural CD103+ cDC1s has been well-described pre-clinically and via their function as the primary producers of the T cell recruiting chemokine CXCL10, they are known to be essential for tumor-homing of effector T cells (63). Furthermore, migratory cDC1s are critical for the continuous trafficking of tumor antigens to the draining LN in a CCR7-dependent process (64, 65).

### Plasmacytoid Dendritic Cells

pDCs are unique in their ability to rapidly produce vast amounts of type I IFNs (IFN-α/β) in response to TLR stimulation (66). In contrast to cDCs, pDCs develop fully in the bone marrow where after they traffic to secondary lymphoid organs (67). Activated pDCs are known to augment CD8+ T cell responses, even in the absence of specific antigen stimulation (68). In addition, studies indicate that TLR-activated pDCs can function as cross-presenting cells (69–71) and even have direct cytotoxic effects (72, 73).

The ability of pDCs to produce vast amounts of type I IFNs upon activation is important for induction of cancer immunity (74, 75). A pre-clinical study reported that tumor rejection was dependent on secretion of type I IFNs (76) and this observation has later been seconded by a study reporting that production of type I IFNs by DCs were required for priming of tumor-reactive T cells and tumor elimination (77). Type I IFNs have been found to promote cross-priming of CD8+ T cells (78), enhance the division of activated CD8+ T cells (79) and stimulate intratumoural accumulation of cross-presenting DCs (80). Stimulating production of type I IFNs by pDCs therefore a feasible strategy to augment tumor-reactivity.

## Strategies to Engage Endogenous DCs For Improved Act Efficacy

Because engagement of DCs alongside ACT is pivotal for broadening the tumor-reactive response, it is relevant to explore treatment strategies that can be used to stimulate DC activation and/or antigen presentation. In this review, the focus will be on pre-conditioning with chemotherapeutics or radiotherapy, peptide or DC-based vaccination, stimulation with toll-like receptor (TLR) agonists or stimulator of interferon genes (STING) agonists, and CD40 ligation as adjuvant treatments to ACT and checkpoint inhibition.

### Pre-conditioning With Chemotherapeutics or Radiotherapy

It is well-established that ACT following lymphodepletion can enhance anti-tumor reactivity in murine and human hosts (81–83), and lymphodepleting pre-conditioning is a standard treatment before T cell infusion in human patients (84). Lymphodepletion refers to an elimination of endogenous lymphocytes, which can be achieved by treatment with a low dose of radiotherapy (RT) or a chemotherapeutic agent, typically cyclophosphamide (CPX) and Fludarabine. Lymphodepletion results in a more pronounced tumor regression than observed with ACT alone and there are several proposed mechanisms behind the improved immunity. Depletion of immune cell subsets that can suppress tumor-reactive T cells, in particular Tregs, has been suggested as an important feature of pre-conditioning before ACT. Pre-clinically, a single treatment with a low dose of doxorubicin or paclitaxel was found to reduce the amount of Tregs and increase the amount of adoptively transferred CD8+ T cells in EG.7-OVA tumors, compared to tumors of mice treated only with chemotherapy or ACT. In this study, it was also found that the frequency of myeloid derived suppressor cells (MDSCs), that can inhibit tumor-reactive T cells through secretion of anti-inflammatory cytokines and amino acid depletion, decreased in the combination treatment groups (85). In a clinical study with metastatic melanoma patients, pre-conditioning with non-myeloablative chemotherapy before adoptive transfer of TILs was likewise shown to decrease the frequency of peripheral CD4+ FoxP3+ Tregs, which in turn correlated with an improved response to the ACT (86). Another proposed mechanism for enhanced ACT efficacy after pre-conditioning is an increased availability of homeostatic cytokines for the infused T cells (87). Under normal physiological circumstances, IL-7, IL-15, and IL-21 exert a supportive role for endogenous T cells because they stimulate homeostatic proliferation. Lymphodepletion, as a result of pre-conditioning, results in a decrease in the endogenous T cell population, which in turn increases the availability of IL-7, IL-15, and IL-21 to support proliferation of adoptively transferred T cells (81).

Enhanced functionality of APCs, in particular DCs, is also believed to play an important role in mediating the enhanced anti-tumor reactivity of adoptively transferred T cells following pre-conditioning (88–90). A pre-clinical study demonstrated that pre-conditioning with CPX enhanced the proliferative capacity of bone marrow-derived DC precursors (91). Compared to untreated control mice, bone marrow harvested from CPX-treated mice generated higher numbers of DCs with the ability to become activated in response to TLR stimulation and prime T cells *in vitro*. These results were in line with previous findings demonstrating that lymphodepletion with a single dose of CPX induced expansion of immature DCs that could be detected in the peripheral blood 8–16 days post treatment (92). Pre-conditioning with CPX followed by a DC based vaccine has also been found to enhance the anti-tumor response in murine hosts, even in the absence of ACT (93), suggesting that CPX stimulates priming of endogenous T cells. Another proposed mechanism, behind the enhanced DC function following pre-conditioning, is induction of immunogenic cancer cell death, which is a result of the anti-proliferative and cytotoxic effects of chemotherapy or ionizing radiation (94–96). Immunogenic cell death involves exposure of several plasma membrane markers that enhances DC functions, e.g., HSP70 and calreticulin that stimulates cross-presentation of tumor derived antigens and phagocytosis, repectively (97). In response to plasma membrane markers associated with immunogenic cell death, DCs become activated and release cytokines, which supports the process of creating a pro-inflammatory environment for T cell priming and activation (98). Immunogenic cell death is also a determining factor for antigen trafficking and presentation by DCs in the tdLN (99). In particular RT is known to induce immunogenic cell death and is therefore a potent DC stimulator. RT also influences APCs directly by inducing cell-intrinsic changes that affect the activation status. This was demonstrated in a study where a single high dose of local RT promoted the activation and functional maturation of a human antigen-presenting cell line through intrinsic DNA damage and p53 activation (100). Also a RT-dependent release of Type I IFNs has been found to be an important contributor to activation of tumor infiltrating innate immune cells, including DCs (101). On the contrary, RT might increase the secretion of TGF-β, which in turn can inhibit the activation and maturation of DCs, and pre-clinical studies have shown that TGF-β blockade enhances the efficacy of RT (102, 103). Negative effects on APCs and consequently the anti-tumor immunity have however also been reported as an effect of RT. In a pre-clinical study, a single dose of RT was found to cause a significant decrease in the amount of CD8+ DCs in murine spleens, which correlated with a switch from a Th1 to a Th2 T cell response (104). In the same study, an analysis of blood samples from RT-treated patients revealed a significant decrease in the frequency of circulating BDCA3+ DCs. Evidently, RT can affect APC function and consequently the anti-tumor immunity in different ways. The main outcome does however seem to be mostly favorable for supporting anti-tumor T cell priming and pre-conditioning using RT or chemotherapy remains to be a well-established therapeutic strategy that enhances the efficacy of ACT.

### Vaccination

Pre-clinical and clinical studies have demonstrated that the *in vivo* expansion, persistence and poly-functionality of infused T cells can be enhanced by providing post-transfer vaccination. Vaccination can facilitate post-infusion priming of adoptively transferred T cells, which stimulates the expansion and functionality and enhances the tumor-reactivity. Clinical trials combining ACT and post-infusion vaccination with viral or tumor antigens are in early stages and with a primary focus on evaluating safety and applicability but encouraging findings have been reported.

Several strategies for enhancing the post-infusion effect of adoptively transferred T cells by vaccination have been explored. One of these is to exploit the ability of viral antigens to stimulate potent T cell activation and expansion, as well as promoting central memory formation. A recent study demonstrated that vaccination with Epstein Barr Virus (EBV) antigen improved the persistence of CD19 CAR T cells modified to recognize EBV in relapsed pediatric acute lymphoblastic leukemia patients (105). In this study, donor Epstein–Barr virus (EBV)-specific cytotoxic T-cells were genetically modified to express a CD19CAR to enhance the long-term persistence of the transferred T cells but also to facilitate a more physiologically relevant expansion of the CARs and avoid cytokine mediated toxicities. An initial patient cohort treated only with the EBV CD19CARs showed poor post-infusion persistence and expansion, but EVB CD19CARs combined with post-infusion EBV vaccination showed enhanced persistence without induction of cytokine release syndrome, neurotoxicity or graft vs. host disease. Another study evaluated the effects of combining a DC-based vaccine with cytomegalovirus (CMV) antigens and CMV-specific T cell transfer in glioblastoma patients (106). CMV-antigens have previously been found to be expressed by GBM which in this study was leveraged to enhance the expansion and persistence of adoptively transferred CMV pp65-specific T cells. For this study, autologous DCs were transfected with CMV pp65 mRNA and infused following adoptive T cell transfer. The results showed that DC vaccination with the CMV pp65 mRNA gave an increase in the frequency of polyfunctional CMV pp65-specific CD8+ T cells with simultaneous expression of IFN-y and TNF-α. This increase in polyfunctional antigen-specific T cells correlated with an improved overall survival.

Stimulating TAA-specific adoptively transferred T cells with post-infusion TAA vaccination has also shown promising results in pre-clinical and clinical trials. Recently, a pre-clinical study found that the persistence and activity of infused CAR-T cells could be enhanced by a tumor antigen vaccination (107). Results from this study showed that the CAR-T cells could undergo post-infusion priming in lymphoid organs which triggered extensive expansion and enhanced anti-tumor efficacy. These results were in line with findings from a previous study demonstrating that a DC-based tumor antigen vaccination significantly enhanced the proliferation, cytokine production and tumor infiltration of infused T cells (108).

Other clinical trials have reported encouraging results related to the objective clinical response of cancer patients treated with the ACT and post-infusion TAA vaccination. In line with these findings, a clinical phase II trial reported an improved 5 year recurrence-free and prolonged overall survival of patients with invasive hepatocellular carcinoma who received a post-operative DC based vaccine combined with ACT (109). This treatment was based on autologous tumor lysate pulsed DCs and transfer of activated T cells.

Recent research has provided evidence of the existence of so-called “anti-regulatory T cells” that recognize immunosuppressive molecules (110, 111). Studies have shown that both human cancer patients and healthy individuals have CD8+ and CD4+ T cells that can respond to factors associated with immune suppression, such as PD-L1 (112), arginase-1 (113), arginase-2 (114), and IDO (115, 116). The existence of anti-regulatory T cells represents an interesting potential of vaccinating against immune suppressive subsets that express these immunomodulatory molecules, e.g., M2 macrophages, MDSCs, and Tregs. Accordingly, an interesting study showed that the immunogenicity of a DC-based vaccine against p53 could be enhanced by co-stimulation with a PD-L1 derived epitope (117). This could potentially be one mechanism to counter-act the inflammation-induced suppressive mechanisms that can counter-act the anti-tumor effects of T cells.

The ability to combine ACT with vaccination, either with viral antigens or TAAs, represents an interesting approach to enhance the efficacy of ACT. The persistence of infused T cells have been linked to therapeutic efficacy (118, 119) and because post-transfer vaccination has been shown to enhance the persistence of infused T cells, future studies should further explore the concept of enhancing ACT with post-infusion vaccination.

### TLR Stimulation

A well-established way to activate DCs is by stimulation with TLR agonists. TLRs are a type of pattern recognition receptors (PRRs) that comprise a group of endosomal and plasma-membrane associated proteins expressed on DCs and other innate immune cells, such as macrophages (120). TLRs are conventionally used as vaccine adjuvants (121) and when DCs or macrophages are stimulated through TLRs, a process of activation and maturation is initiated which results in secretion of T cell activating cytokines such as TNF-α, IL-6, IL-12, and type I IFNs (122). To this date, three TLR agonists have been approved by US regulatory agencies to treat cancer patients; (1) Imiquimod, a TLR7 agonist used to treat superficial basal cell carcinoma, (2) Bacillus Calmette-Guérin (BCG), supposedly stimulating TL2, TLR3, and TLR9, used to treat non-invasive transitional cell carcinoma of the bladder, and (3) monophosphoryl lipid A (MPL) stimulating TLR4 and used in a prophylactic vaccine against HPV-virus (123).

TLR stimulation can be used in combination with tumor antigen delivery to ensure activation of antigen presenting DCs. TLR stimulation can however also be used as an adjuvant treatment to other cancer therapies that induce tumor antigen release, such as RT. The combination of TLR stimulation and RT has been explored in pre-clinical studies with encouraging results. A study reported that intravenous administration of the TLR7 agonist resiquimod (R848) in combination with RT lead to clearance of established tumors in murine lymphoma models (124). The treatment effect was associated with expansion of tumor-antigen specific CD8+ cells and improved survival of the treated mice. These results were in line with findings from other pre-clinical studies demonstrating that TLR7/8 agonists can be potent adjuvants to RT by boosting antigen-presentation by DCs in subcutaneous and orthotopic mouse models of colorectal and pancreatic cancer (125). Similarly, systemic administration of a TLR7 agonist in combination with RT has been shown to prime a tumor-reactive CD8+ T cell response and result in improved survival in syngeneic models of colorectal carcinoma and fibrosarcoma (126). The combination of RT and TLR stimulation is interesting because the RT-induced availability of tumor antigens at the tumor site can be exploited to stimulate an endogenous tumor response. RT acts directly on cancer cells and introduce DNA damage that, if left unrepaired, results in cell death (95, 96). This can stimulate an anti-tumor T cell response, when APCs engulf (parts of) dying cancer cells for subsequent T cell priming. In this setting, TLR stimulation can feed into the circle of events and boost activation and antigen-presentation of DCs that enhances the T cell priming.

To the best of our knowledge, no clinical trials evaluating the effect of enhancing ACT efficacy by TLR stimulation have so far been completed. Pre-clinical studies have however described the use of TLR stimulation to augment ACT with encouraging results. A recent study reported that the administration of the TLR4 agonist Lipopolysaccharid (LPS) could augment the tumor-reactivity of adoptively transferred pmel-1 CD8+ T cells in mice with established B16.F10 tumors (127). Administration of MPL and the TLR9 agonist CpG ODN likewise potentiated the anti-tumor activity of infused CD8+ T cells. These results were in line with findings from a previous study demonstrating that TLR3 stimulation and a tumor antigen vaccination increased the expansion and anti-tumor efficacy of adoptively transferred antigen-specific pmel-1 CD8+ T cells in pre-conditioned B16.F10 tumor-bearing mice (92).

Although TLR agonists are effective vaccine adjuvants, the overall beneficial effect of TLR stimulation as cancer immunotherapy is debated. The concept is complicated by the fact that TLRs are not only expressed by immune cells but also by cancer cells (128) and TLR expression by cancer cells has been linked to metastasis in breast cancer (129) and esophageal squamous cell carcinoma (130). Accordingly, the reported effects of TLR treatment as a cancer immunotherapy have been mixed (123, 131), reflecting issues with toxicity and supposedly the complexity of TLR expression in the tumor microenvironment. Therefore, additional research is needed to further elucidate the effect of TLR stimulation as a cancer immunotherapy and current issues related to safety and administration of TLR agonists have to be resolved.

### STING Agonists

Given the importance of type I IFNs in cancer immunity, efforts have been put into identifying pathways that are responsible for or linked to secretion of type I IFNs. Recent findings have pointed toward a crucial role for the STING pathway in this process (132). STING is an adapter molecule that becomes activated by cyclic dinucleotides generated by cyclic GMP-AMP synthase, which in turn is activated by cytosolic DNA. Activated STING phosphorylates interferon regulatory factor 3 (IRF3) that directly contributes to type I IFN gene transcription (133). One study found that spontaneous tumor-reactive CD8+ T cell priming was defective in STING^−/−^ mice and that STING pathway activation and IFN-β production correlated with DNA detection in tumor infiltrating DCs (134). The STING pathway therefore appears to be involved in detecting the presence of a tumor to drive DC activation and subsequent T cell priming against tumor associated antigens.

STING agonists have been shown to have therapeutic implications for stimulating anti-tumor T cell reactivity. Pre-clinical studies have described that treatment with STING agonists can induce an increase in the abundance and functionality of tumor-infiltrating cytotoxic T cells associated with tumor regression (135–137) and STING stimulation has also been shown to antagonize expansion of immune suppressive myeloid derived suppressor cells (138). Interestingly, a pre-clinical study recently demonstrated that co-delivery of a STING agonist and CAR-T cells resulted in elimination of tumor cells that were not recognized by adoptively transferred CAR-T cells as monotherapy (139). The combined delivery of a STING agonist and CAR-T cells resulted in a synergistic activation of APCs and was associated with prolonged survival and protection against tumor escape. These results indicate that STING activation might enhance the effect of CAR-T cell therapy by broadening the tumor-reactive T cell response.

Given the ability of STING to stimulate priming of tumor-reactive T cells through DC activation, it is likely that STING activation can enhance epitope spreading when combined with ACT. The combination of STING and checkpoint inhibitors is being tested in clinical settings and has been shown to enhance the therapeutic response to chemotherapy in patients with ovarian cancer (140). A study done in a pre-clinical model of head and neck cancer also demonstrated that the combined treatment of STING and PD-1 blockade could enhance local and systemic immunity and reverse adaptive resistance to chemotherapy (141). The ability to (re)establish tumor immunity using STING and checkpoint inhibition indicates that STING activation can expand pre-existing tumor reactivity, perhaps by broadening the tumor response. STING activation could thereby support the therapeutic efficacy of adoptively transferred T cells by activating APCs that in turn can cross-prime endogenous tumor-reactive T cells.

### CD40 Stimulation

Another well-established mechanism, which is implicated in priming of tumor-reactive T cells, is engagement of the CD40/CD40L axis. CD40 is a member of the Tumor necrosis factor (TNF) receptor family and is expressed by a range of different cell types including DCs, B cells, platelets and non-hematopoietic cells such as endothelial cells, fibroblasts and some types of cancer cells (142). Ligation of CD40 on DCs has been found to upregulate the expression of co-stimulatory molecules (e.g., CD80 and CD86) and MHC molecules, induce secretion of pro-inflammatory cytokines and enhancing the antigen processing machinery (143). Stimulation with CD40 agonists has been shown to stimulate the T cell priming abilities of DCs and lead to potent anti-tumor T cell immunity in pre-clinical models. Early mechanistic studies also demonstrated that CD40 stimulation can enhance the efficacy of tumor antigen vaccination (144, 145), induce activation of endogenous CD4+ T cells (144) and reverse cytotoxic T cell tolerance (145). Interestingly, pre-clinical studies indicate that activated CD8+ T cells are able to boost IL-12 production by DCs through expression of CD40L. Consequently, this could provide a positive feedback loop for adoptively transferred T cells through the engagement of DCs (146).

The ability of CD40 stimulation to enhance the efficacy of ACT has been evaluated in different pre-clinical models. Recently, a study showed that lymphodepleting WBI in combination with CD40 stimulation enhanced the accumulation of infused T cells in a murine pancreatic tumor model (147). Here it was demonstrated that the combination of CD40 stimulation, WBI and ACT enhanced the proliferation of infused T cells, promoted high levels of tumor inflammation and was associated with tumor regression and prolonged survival of treated mice. These findings were in line with observations from a previous study, where CD40 stimulation was shown to boost the anti-tumor activity of ACT in murine B16.F10 tumors (148). Here, a monoclonal antibody targeting CD40 was combined with ACT and administration of IL-2. This combination improved the expansion of the infused T cells and was associated with tumor regression. The results also showed that the T cell expansion was dependent on IL-12 and expression of CD80 and CD86 by endogenous DCs.

The use of CD40 agonists has also been tested as cancer immunotherapy in clinical settings. A paper recently summarized the long-term outcomes of a phase I study of agonistic CD40 antibody and CTLA-4 treatment of metastatic melanoma patients (149). Here it was found that the therapy was associated with increased tumor T cell infiltration, T cell reinvigoration and T cell clonal expansion. The positive effects of CD40 stimulation on T cell priming can however not necessarily be attributed to DC stimulation alone. Co-stimulatory signaling through CD40/CD40L interaction influences not only DCs but also macrophages and in particular B cells. CD40 stimulation promotes the survival and expansion of B cells and supports their development into antibody-secreting plasma cells and memory B cells. CD40 stimulated B cells also undergo somatic hyper-mutation of the Ig, which gives and enhanced antigen-affinity (150). Importantly, since B cells can also function as APCs, it is possible that the enhanced T cell functions as a result of CD40 stimulation can be attributed to the effects of both activated B cells and DCs. Collectively, findings from pre-clinical and clinical studies indicate that CD40 agonists, via the ability of CD40 stimulation to enhance APC functions and T cell priming, could be a feasible adjuvant therapy to ACT.

## Discussion

The post-infusion performance of adoptively transferred T cells is critically dependent on several factors, in particular the composition of the TME that can favor or suppress the tumor-reactivity of the T cells. Studies have also linked the post-infusion performance to the differentiation status of the adoptively transferred T cells, with less differentiated T cells, such as naïve or stem cell memory T cells, having greater capacity for expansion and persistence after transfer. The T cells used for ACT typically have a more differentiated effector T cell phenotype at the time of infusion, because the *in vitro* expansion step of the process involves activating and stimulating T cell proliferation to reach a large number of cells. This activation and stimulation of T cells induce a stepwise differentiation process, which ultimately leads to generation of T cells with a terminally differentiated and short-lived effector phenotype that is suboptimal for ACT (151). Because T cells with a less differentiated phenotype have greater post-infusion expansion and persistence potential (10, 12), efforts are being put into designing strategies to maintain the less differentiated phenotype of T cells used for ACT. These strategies include optimizing the T cell culture conditions to preserve a less differentiated phenotype during expansion. This can be done e.g., by adding specific (combinations of) cytokines (152, 153), small molecule inhibitors (154, 155) or co-stimulatory molecules (156, 157) to the culture medium. Another strategy is to generate antigen-specific T cells that are induced from pluripotent stem cells (158, 159). The less differentiated T cells are however critically dependent on post-infusion priming with their cognate antigen to have any tumor-reactivity, which can be provided through endogenous DC stimulation. Therefore, if T cells with e.g., a stem cell memory phenotype are being used for ACT, it could be favorable to combine the T cell infusion with post-infusion vaccination with the cognate antigen of the T cells. If T cells with a more differentiated phenotype, such as effector memory, are being infused, the secretion of pro-inflammatory cytokines might be sufficient for enhancing the post-infusion expansion and persistence. When choosing the optimal add-on therapy to ACT, it is therefore important to take into consideration the differentiation status of the infused T cells.

In addition to the examples given above, another emerging strategy is to modify the T cell product prior to infusion, thereby “arming” the T cells with the ability to engage endogenous DCs. Recent pre-clinical studies have investigated ACT with genetically modified T cells expressing membrane-anchored IL-12 (160), which is known to increase co-stimulation by dendritic cells. In addition, T cells engineered to express the DC-recruiting cytokine FLT3L, was recently shown to enhance efficacy and support epitope spreading after ACT therapy (161). With the advancements made within engineering of T cells for ACT, it is feasible to include the DC-priming strategy—not only as an add-on treatment, but also as an intrinsic property of the transferred T cells.

## Concluding Remarks

The concept of ACT has been manifested as a promising therapeutic option for a subgroup of patients with melanoma and hematological malignancies. To further improve the therapeutic efficacy of ACT, and to broaden the application to other types of solid cancers, it is necessary to expand our knowledge on factors that can enhance the post-infusion persistence and functionality of transferred T cells. Engagement of DCs, as an adjuvant therapy to ACT, can stimulate a broader tumor-reactive response by priming endogenous T cells and facilitate post-infusion priming of adoptively transferred T cells. Accordingly, the combination of ACT and DC-activating treatments such as TLR or STING agonists, as well as CD40 stimulation and vaccination with viral or tumor antigens, has been found to have implications for the *in vivo* expansion, persistence and polyfunctionality of infused T cells. Engagement of activated DCs alongside ACT has also been associated with improved tumor control and prolonged survival in pre-clinical models. The addition of checkpoint blockade alongside ACT and DC stimulation can be utilized to counteract an eventual inactivation of tumor-reactive T cells and might provide an additional synergistic effect. The combination of ACT, treatments that activates and/or induces antigen presentation of DCs and checkpoint blockade therefore represents an interesting therapeutic strategy that potentially can enhance the efficacy and broaden the applicability of ACT in the future.

## Author Contributions

MH and DJ did the literature search and wrote the review. TA and MA revised and approved the final version of the review. All authors contributed to the article and approved the submitted version.

## Conflict of Interest

The authors declare that the research was conducted in the absence of any commercial or financial relationships that could be construed as a potential conflict of interest.
